# Low divergent MeV-class proton beam with micrometer source size driven by a few-cycle laser pulse

**DOI:** 10.1038/s41598-022-12240-2

**Published:** 2022-05-16

**Authors:** Prashant K. Singh, Parvin Varmazyar, Bence Nagy, Joon-Gon Son, Sargis Ter-Avetisyan, Karoly Osvay

**Affiliations:** 1 National Laser-Initiated Transmutation Laboratory, University of Szeged, 6720 Szeged, Hungary; 2 ELI-ALPS Research Institute, ELI-HU Non-Profit Ltd, Wolfgang Sandner utca 3, 6728 Szeged, Hungary; 3 Department of Optics and Quantum Electronics, University of Szeged, 6720 Szeged, Hungary

**Keywords:** Plasma-based accelerators, Laser-produced plasmas

## Abstract

Spatial characterization of 0.5 MeV proton beam, driven by 12 fs, 35 mJ, 10^19^ W/cm^2^ intense laser-foil interaction is presented. The accelerated proton beam has been applied to obtain a high-resolution, point-projection static radiograph of a fine mesh using a CR-39 plate. The reconstruction of mesh edge blurring and particle ray tracing suggests that these protons have an effective source size (FWHM) of just 3.3 ± 0.3 µm. Furthermore, the spatial distribution of the proton beam recorded on the CR-39 showed that the divergence of these particles is less than 5-degree (FWHM). The low divergence and small source size of the proton beam resulted in an ultralow transverse emittance of 0.00032 π-mm-mrad, which is several orders of magnitude smaller than that of a conventional accelerator beam.

## Introduction

Intense femtosecond laser-driven proton beams^1,2^ have unique properties, both in the temporal (picosecond bunch duration^3^) and in the spatial domain (small source size^4–6^ and laminar beam^7^). Thanks to these features, the laser-driven proton beams can be potentially used in a wide range of applications such as unravelling the transient plasma-dynamics via proton imaging^8,9^, creation of warm-dense-matter state by isochoric heating of solid materials^10^, radiobiological effects of laser-driven ions^11,12^ or transmutation^13,14^. Some of these applications, due to high-flux requirement^10,11,13,14^, may be benefited from a collimated particle beam. This is where the laser-driven ions suffer due to inherent large angular divergence (typically in the range of 10°–60°)^15,16^. To improve the beam collimation, several external, post-acceleration schemes^17–20^, involving both static (using quadrupole magnets^21,22^) and dynamic lensing^23,24^ have been demonstrated. Recently, few experiments have improved the inherent laser-driven proton divergence after suppressing the laser prepulse with double-plasma mirrors^25^ or with saturable absorbers^26^. These studies suggest that a laser system with high-temporal intensity contrast could be more effective in achieving a low divergent proton beam. Reduction of the inherent beam divergence can also be beneficial in minimizing particle flux losses that occur during the post-acceleration beam guiding phase^21,22^. Besides the issue of beam collimation, some applications^8,9^ also demand a small source size, as this affects the ultimate spatial resolution that can be realized in point-projection radiography^5^ or ion-beam lithography^27^. Unlike the beam collimation^21,22^, improving the inherent proton source size is non-trivial, and much harder to be corrected by post-acceleration schemes^28^. The combined effect of these two features, namely, the beam collimation and small source size can help in minimizing the transverse emittance of the beam, which defines the merit of beam transport^29^ and ultimate focal spot^7^. Besides promising aspects of improvement in the beam divergence and source size, the inherent broadband spectrum of the laser-driven proton beam remains a concern while considering the transport or post acceleration of such a beam. Reducing the energy spread of laser-driven ion beams has been a long-standing goal, which requires better understanding and control of complex underlying physics of laser-solid interaction^1^.

So far, high-energy, multi Joule level laser systems, operating under single-shot mode have been used to drive these experiments^1,2^. Recently, a different regime of generating high-quality particle beams has been explored with modest energy (10’s mJ), few-cycle laser systems^30–34^. By focusing a few-cycle, mJ laser pulse to the diffraction limit, relativistic intensities^35^ or the so-called lambda-cube regime^36^ can be achieved. These systems, operating at kHz^37,38^ or higher repetition rate, can generate MeV proton beams^32,33^ and could provide the users with a similar average particle flux (although at much lower ion cut-off energy) compared to what multi-Joule laser systems on a single shot basis do. These MeV-class proton beams can find direct applications in areas such as ion-beam implantation^39^, ion injector in a conventional accelerator^40,41^, production of bright neutron flux via D(d, n) reaction^42–45^ for transmutation of spent nuclear fuel^14^ or recreation of rapid (r) process for nucleosynthesis^46,47^. Towards these goals, here we present the spatial characterization of a laser-driven proton beam generated at an intensity of 10^19^ W/cm^2^ with a 12 fs laser pulse. Our measurement with point-projection imaging technique^4,6^ shows that accelerated proton beams of 0.5 MeV energy have effective source size as small as 3.3 µm and are well confined with an angular divergence of less than 5°. Due to low-divergence and small source size, these proton beams possess ultra-low transverse emittance and therefore can be used for efficient particle transport or as an injector in the accelerators.

## Results

### Experimental set-up

**Figure 1:**
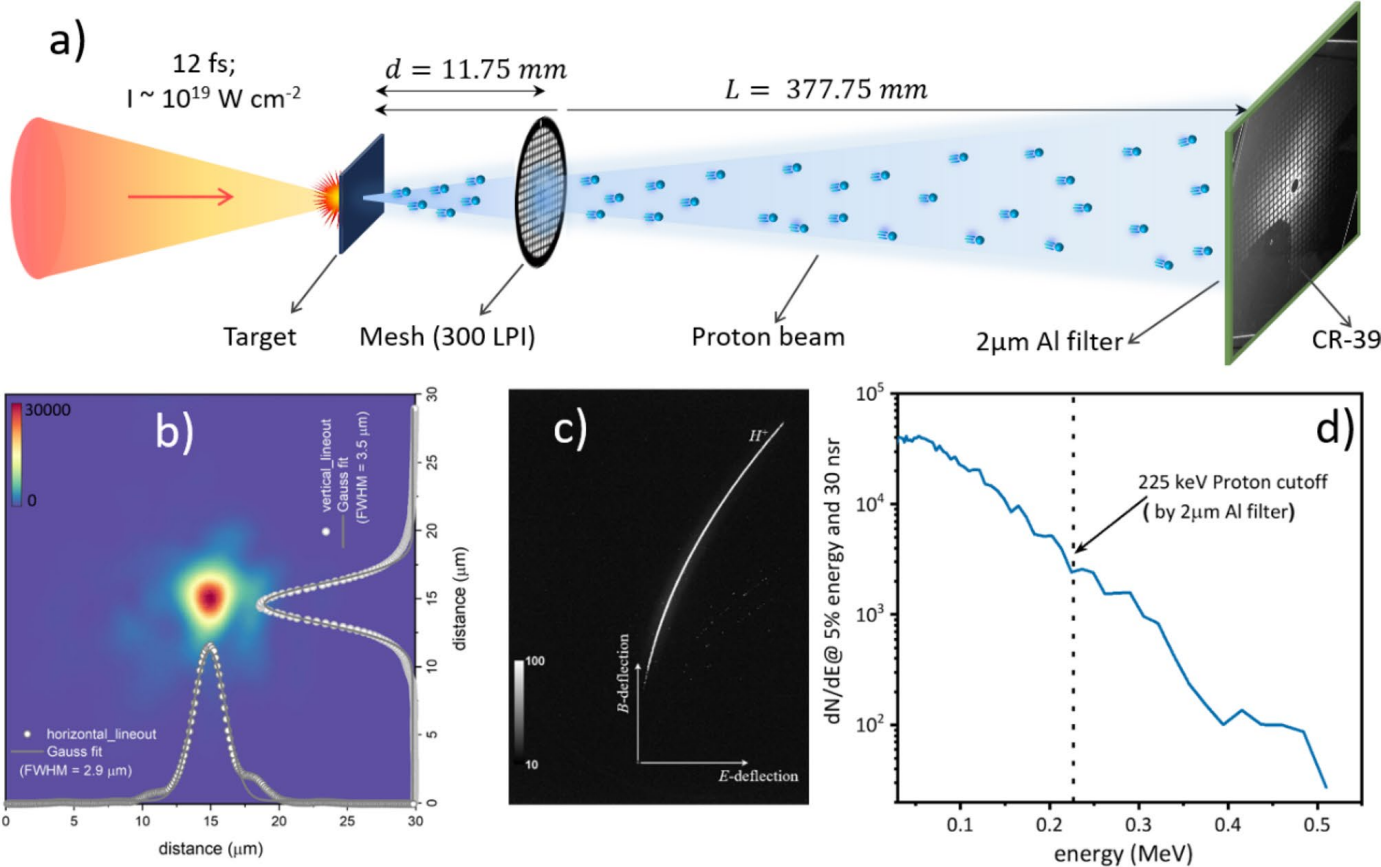
Generation of laser-driven proton beam for point-projection imaging. (**a**) Schematic of the experimental set-up. A linearly polarized, 12 fs laser pulse irradiate the target (2 µm thick Al foil) at zero angle of incidence with a peak irradiance of 10^19^ W/cm^2^. A magnified image (32.2×) of the 300 lines-per-inch (LPI) mesh is projected on the CR-39 plate using the accelerated proton beam. (**b**) Measured laser focus intensity distribution along with horizontal and vertical line profile, fitted with gauss function having FWHM of 2.9 µm and 3.5 µm, respectively. (**c**) Raw TPS data, showing the dominance of proton beam over other ions and (**d**) corresponding calculated kinetic energy spectra of the proton.

The experiment was performed with the SYLOS Experimental Alignment Laser (SEA Laser) at the ELI-ALPS facility in Szeged, Hungary^38^, details described in the method section. Briefly, the laser-driven proton beam was generated by a 12 fs, 35 mJ, linearly polarized, NIR (840 nm) laser pulse, focused on a 2 µm thick Al foil target at zero angle of incidence (Fig. 1a). The measured laser focal spot of 2.9 µm $\times$ 3.5 µm (FWHM), lead to the estimated peak intensity of 10^19^ W/cm^2^ (Fig. 1b). The kinetic energy spectrum of the forward accelerated proton beam was measured with a Thomson Parabolic spectrometer (TPS). Figure 1c shows the raw image of the particle traces, where protons dominate over other ion species such as carbon or oxygen. The corresponding estimated kinetic energy spectrum (Fig. 1d) of the proton beam shows cut-off energy of 0.5 MeV.

The spatial characterization of the proton beam was carried out by projection imaging of a periodic mesh with a proton beam accelerated by a single laser shot onto a CR-39 plate. The geometric magnification of the mesh, calculated from the distance (Fig. 1a), is 32.2. The 300-LPI Copper mesh, having a thickness of 20 µm, blocks protons up to 2 MeV (higher than our proton cut-off energy of 0.5 MeV) and therefore ensure no possible degradation of edge sharpness due to partial transmission of protons while crossing the mesh. To avoid the saturation of the CR-39 plate from the low energy protons and the Carbon ions, a 2 µm thick Al filter was used in front of the CR-39. This filter can block protons up to 225 keV (shown by the vertical dash line in Fig. 1d) and carbon ions up to 1.8 MeV, which is much higher than the measured carbon ions during the experiment.

### Angular divergence of the proton beam

**Figure 2:**
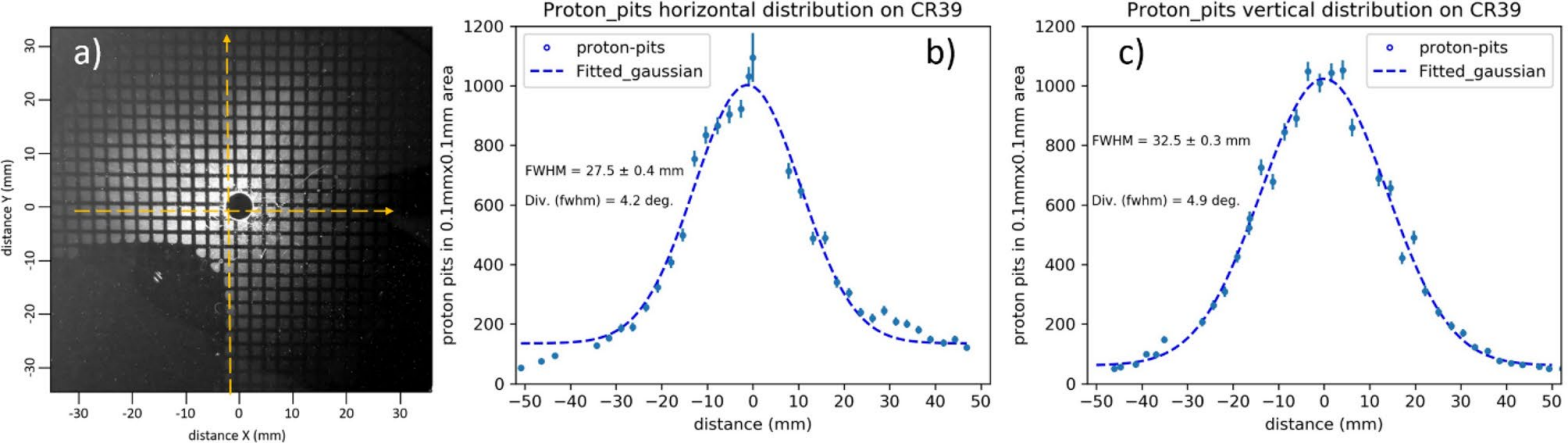
Spatial distribution and angular divergence of proton beam. (**a**) Image of magnified 300 LPI mesh recorded on CR-39 by protons (energy > 225 keV). (**b**) The spatial distribution of the proton pit across the CR-39 plate, taken along the horizontal (**b**) and vertical (**c**) dash line shown in Fig. (**a**). Each data point for proton distribution is sampled over an area of 0.01 mm^2^. The proton pit distribution data are fitted with a Gaussian function.

The proton beam spatial distribution, captured on the exposed CR-39 plate (Fig. 2a), shows that accelerated protons are well localized and centered along the target normal direction. Proper selection of the Al filter (2 µm thick) and distance of CR-39 from the target ($\sim$ 38 cm) ensured that the proton pit number density (pits/mm^2^) were statistically robust and at the same time overlapping of proton pits or saturation of CR-39 plate was avoided. The spatial distribution of the proton beam has been plotted along two orthogonal directions, the horizontal (Fig. 2b) and the vertical (Fig. 2c), as indicated by yellow dash lines in Fig. 2a. The proton count density (${n}_{p}$) in the central axis peaked at about 1100 pits in 0.01 mm^2^ area whereas 50 mm away from the central axis it dropped below 100 pits per 0.01 mm^2^ area. The error bar in the data represents the variation of counts by sampling from different regions of the same area of 0.01 mm^2^ and standard deviation ($\sqrt{{n}_{p}}$). The horizontal and vertical proton spatial distribution, fitted with a Gaussian function, shows a FWHM of 27.5 mm and 32.5 mm, respectively, which corresponds to the proton angular divergence of 4.2$^\circ$ (FWHM) and 4.9$^\circ$ (FWHM) along the horizontal and vertical axis, respectively. This indicates that in our experimental conditions, the accelerated proton beam is low-divergent in nature.

### Proton source-size and emittance estimations

**Figure 3:**
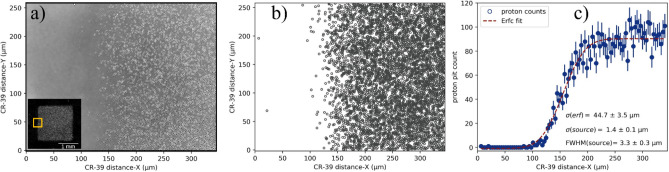
Spatial resolution and effective source size of proton beam. (**a**) 50× Magnified image of the proton pit distribution across a mesh edge and the corresponding cell (inset figure) of the 300 LPI mesh recorded on CR-39 by protons (energy > 225 keV). (**b**) Reconstructed proton pit image by numerical cell detection technique. (**c**) The proton pit distribution along the horizontal axis, with counts summed along the vertical axis. To determine the edge sharpness, the proton pit distribution data are fitted with an error function.

The effective source size or the spatial resolution of the low-divergent proton beam is explored by examining the sharpness of the mesh edge. A 5× magnified image of the central mesh cell capture on the CR-39 is shown in the inset of Fig. 3a, with sharp edges on all four sides. To quantify the sharpness of the mesh edge, a 50× magnified image is recorded (Fig. 3a) along the left edge of the central cell, indicated by the Yellow box in the inset Fig. 3a. To determine the proton pit distribution, a numerical cell detection technique has been used, shown as reconstructed pit image in Fig. 3b. The deviation of pit counts between the numerical technique and manual counting was found to be less than 5%. The distribution of proton pit count along the horizontal axis (*x*), with summed along the vertical axis (y) is displayed in Fig. 3c. The proton counts starting from the baseline of nearly zero counts, sharply rises to a flat level of $\sim$ 100 counts. Here, just like in Fig. 2, the error bar represents the standard deviation ($\sqrt{{n}_{p}}$) of measured proton counts.

An extended, finite-size proton source leads to penumbral blurring of the mesh edge in the CR-39 plane. The mesh edge blurring can be considered as a convolution of proton source distribution at its origin and the edge transmission^48^. Considering step-function for the mesh edge and Gaussian distribution of proton source, the mesh edge blurring can be modelled with Gauss error function whose width corresponds to source width. After fitting the proton edge distribution with the error function (Fig. 3b,c), the width of the error function (${\sigma }_{erf}$) in the detector plane for left edge is 44.7 ± 3.5 µm, which in the target source plane corresponds to source size (${\sigma }_{source}$) of 1.4 ± 0.1 µm and FWHM = 3.3 ± 0.3 µm.

The measured value of proton divergence (Fig. 2) and the derived source size (Fig. 3) can be used to estimate the normalized emittance (${\varepsilon }_{nt}$) of the proton beam, given as ${\varepsilon }_{nt}= \beta \gamma {\sigma }_{r}{\sigma }_{\theta }$, where $\beta$ and $\gamma$ are the proton beam Lorentz factors, ${\sigma }_{r}$ proton RMS source size in mm and ${\sigma }_{\theta }$ is proton RMS divergence in mrad^48^. For our 0.5 MeV proton beam, applying $\beta =0.02302$, $\gamma =1.00053, {\sigma }_{r}=0.0014\, {\mathrm{mm}}, {\sigma }_{\theta }=30.89 \,{\mathrm{mrad}}$, the value for normalized emittance is estimated to be (${\varepsilon }_{nt}$) = 0.00032 $\uppi \,{\mathrm{mm}}\,{ \mathrm{mrad}}.$ The emittance value estimated here relies on two experimental parameters, namely the beam divergence and proton source size. Here, the proton beam divergence was directly measured by the two-dimensional distribution of particles recorded on the CR-39 detector (Fig. 2a). However, the proton source size was indirectly inferred by observing the mesh edge blurring (Fig. 3) and by ballistic ray tracing of proton beamlets (Fig. 5). Both of these methods are based on static projection imaging, and therefore the emittance estimation shown here cannot capture any dynamics involved in the proton source. Previously reported emittance value of laser-driven MeV protons from the mesh projection (or ‘pepper-pot’) methods are about 0.1 $\uppi \,{\mathrm{mm}} \,{\mathrm{mrad}}$^4,49^. Other measurements using mesh projection have recently reported emittance values of 0.01 π mm mrad^50^ and 0.065 π mm mrad^6^. In alternate methods of micromachining groves at the target rear surface, the transverse emittance for nearly 10 MeV proton came to about 0.0013 $\uppi \,{\mathrm{mm}}\,{\mathrm{mrad}}$^7,15^, i.e., 100-fold better than conventional accelerators.

### Location of proton source in point-projection imaging

**Figure 4:**
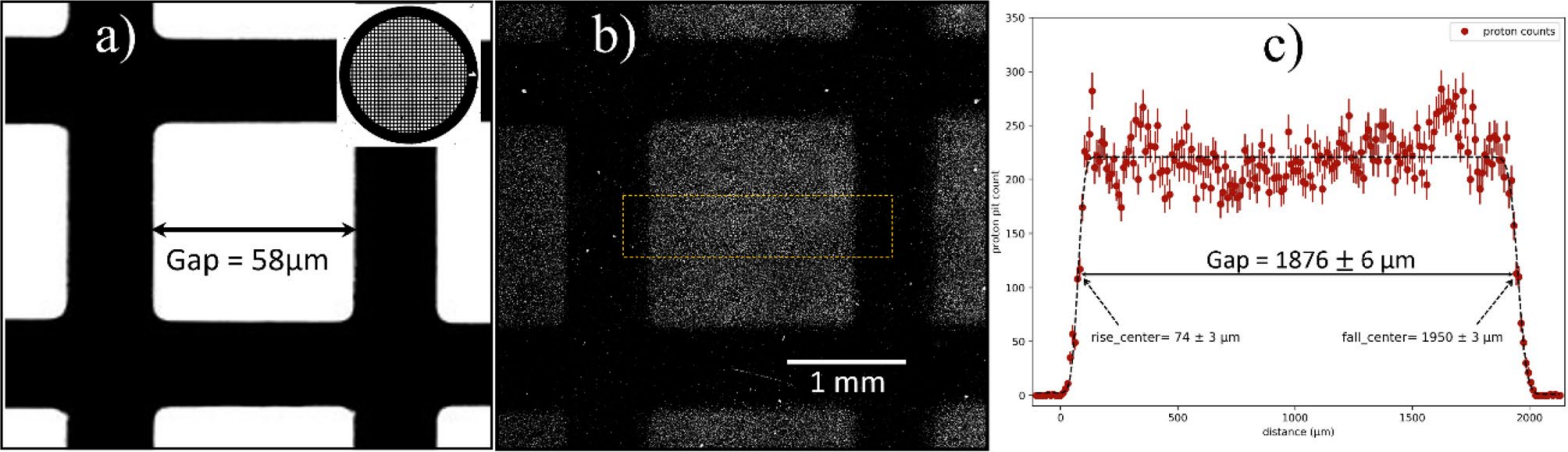
Geometrical versus measured magnification factor. (**a**) Zoom image of the hole region (with a gap of 58 µm) present in the 300 LPI mesh pattern. The full 300 LPI mesh picture shown in inset of (**a**). (**b**) Point-projection imaging of the hole region recorded by the CR-39 plate. (**c**) The spatial distribution of the proton pit count across the dash yellow rectangle in (**b**), showing a sharp rise, middle saturation region, and sharp fall. To determine the beginning and end location of the hole image, the rising and falling slopes are fitted with the Gauss-error function.

In the context of point-projection imaging, it is interesting to see how the experimentally measured magnification factor (${M}_{exp}$) and the expected geometrical magnification factor (${M}_{geo}=L/d =(377.8 \pm 0.5 \,{\mathrm{mm}})/\left(11.750 \pm 0.005 \,{\mathrm{mm}}\right)= 32.15 \pm 0.05$) relate to each other (Fig. 1a). For the calculation of the magnification factor, one of the hole areas (58 µm) of the 300 LPI mesh is chosen (Fig. 4a). The point projection image of the 58 µm hole, recorded on the CR-39 detector is displayed in Fig. 4b). The spatial distribution of the proton pit count across the cell shows two sharp side mesh edges and a broad middle plateau region (Fig. 4c). The measured hole size, after fitting the rising and falling slopes with Gauss-error function turns out to be $1876 \pm 6$ µm (Fig. 4c) and hence the experimental magnification factor (${M}_{exp}= (1876 \pm 6)/\left(58\right)= 32.3 \pm 0.1$). This indicates that here the measured magnification differs just 0.3% from the expected geometry or in other words, the ray-tracing of particle beamlets maps to plane in the vicinity of the target foil, from where the protons are accelerated.

**Figure 5:**
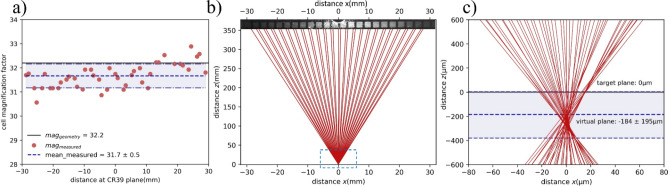
Ray-tracing of proton beamlets. (**a**) Distribution of the measured magnification factor by CR-39 detector for different cells of 300 LPI mesh. The black-horizontal line shows an expected magnification factor of 32.2 from ideal point-projection imaging. The blue-dash line shows the average value of the magnification factor measured within one-standard deviation (± σ). (**b**) Ray-tracing of the proton beamlets drawn by joining the location of mesh edge points in the plane of CR-39 detector (*z*_*CR39*_ = 377.8 mm) to the corresponding points in the 300 LPI mesh plane (*z*_*mesh*_ = 11.75 mm). The top panel in (**b**) shows the picture of the magnified mesh grid recorded on CR-39. (**c**) Zoom image of the highlighted area (indicated by blue dash rectangle in **b**) of proton beamlets close to the target plane (*z*_*target*_ = 0.0 mm). The extent of the possible plane of the virtual source, estimated from the measured magnification factors in Fig. **a**, is highlighted by the blue color.

Previous point-projection proton radiographs, obtained with low or medium temporal contrast lasers, have found that the ${M}_{exp}$ can differ significantly from ${M}_{geo}$ by as much as 60–70% and the ray-tracing of proton beamlets lead to a location sub-mm far from the actual target location^4,49^. The mismatch between magnification factors in the point-projection radiographs was explained with a concept of virtual source^4,6,7^. Considering ballistic, straight-line trajectories of the proton beam, the virtual source corresponds to the point where the ray tracing forms a minimum waist^7^. The virtual source location ($x$) from the actual target position can be determined from the observed magnification (${M}_{exp}= (x+L)/(x+d)$) in the experiment, which was smaller than the expected geometric magnification (${M}_{geo}= (L)/(d)$), where $d$ and $L$ are distances from the target to the mesh and the target to the detector, respectively^4,49^. To estimate the virtual source location ($x$) from the actual target, a detailed mapping of the ${M}_{exp}$ factor was carried out across the vertical axis of the CR-39 (yellow dash arrow in Fig. 2a). To obtain the ${M}_{exp}$ factor, the cell period measured on the CR-39 plane was divided by the 83 µm cell periodicity of the 300 LPI mesh, results shown in Fig. 5a. The average value of measured magnification $\langle{M}_{exp}\rangle =31.7$ is found to be slightly smaller than the geometrical magnification factor ${M}_{geo}=32.2$, considering the one sigma deviation of measured data points (σ = 0.5), area shaded by the blue color in Fig. 5a). This indicate that the origin of the proton beamlets could lie beyond the target plane hence resulting in lower magnification. For better visualization of the proton source, the point-projection technique was used in the backward direction for ray-tracing of the proton beamlets, connecting points in the CR-39 detector plane (*z*_*CR39*_ = 377.8 mm) to the mesh plane (*z*_*mesh*_ = 11.75 mm), as shown in Fig. 5b). These beamlets can be further extended towards the target plane to examine virtual source size and its location.

On a few mm spatial scale (Fig. 5b), the proton beamlets appear symmetric, uniformly distributed and tend to get merged at *z* = 0 mm plane. A magnified view of the beamlets (region indicated by blue dash rectangle in Fig. 5b) reveals that the rays merge beyond the target plane, as expected from the lower measured magnification factor (Fig. 5a). Moreover, looking at the merging and crossing trajectories of the rays, a plane of minimum beam waist can be determined. For instance, the D_80_ diameter^51^, encompassing 80% of the beamlets is estimated to be D_80_ = 3.5 µm at the plane of *z* = − 250 µm. This estimated beam waist of the virtual source is in good agreement with the spatial resolution of the proton imaging realised in the experiment while looking at the mesh edge blurring (FWHM = 3.3 µm, Fig. 3c).

Radiograph imaging with a perfectly laminar proton beam will have a step-like edge response unless restricted by the detector spatial resolution. In the present study, the use of a CR-39 detector allows one to examine the edge response down to 30 nm resolution in the source plane (considering magnification factor of 32 and spatial resolution of the CR-39 detector down to sub-micrometre scale, primarily limited by the size of proton pits). The results obtained here indicate that the laser-driven proton beams are quasi-laminar, where some proton beamlets are crossing each other near the target plane (Fig. 5c), and therefore a finite, measurable edge blurring of ${\sigma }_{erf}= 44.7 \pm 3.5\,\upmu{\mathrm{ m}}$ was obtained (Fig. 3c). It has to be the emphasised here that the visualization of the proton source drawn here is mainly coming from a static projection imaging diagnostic, and hence is capable of reproducing scenario with assumption that these particles retain ballistic trajectories throughout their journey from source to the detector plane.

### Synthetic point-projection imaging with Geant4

**Figure 6:**
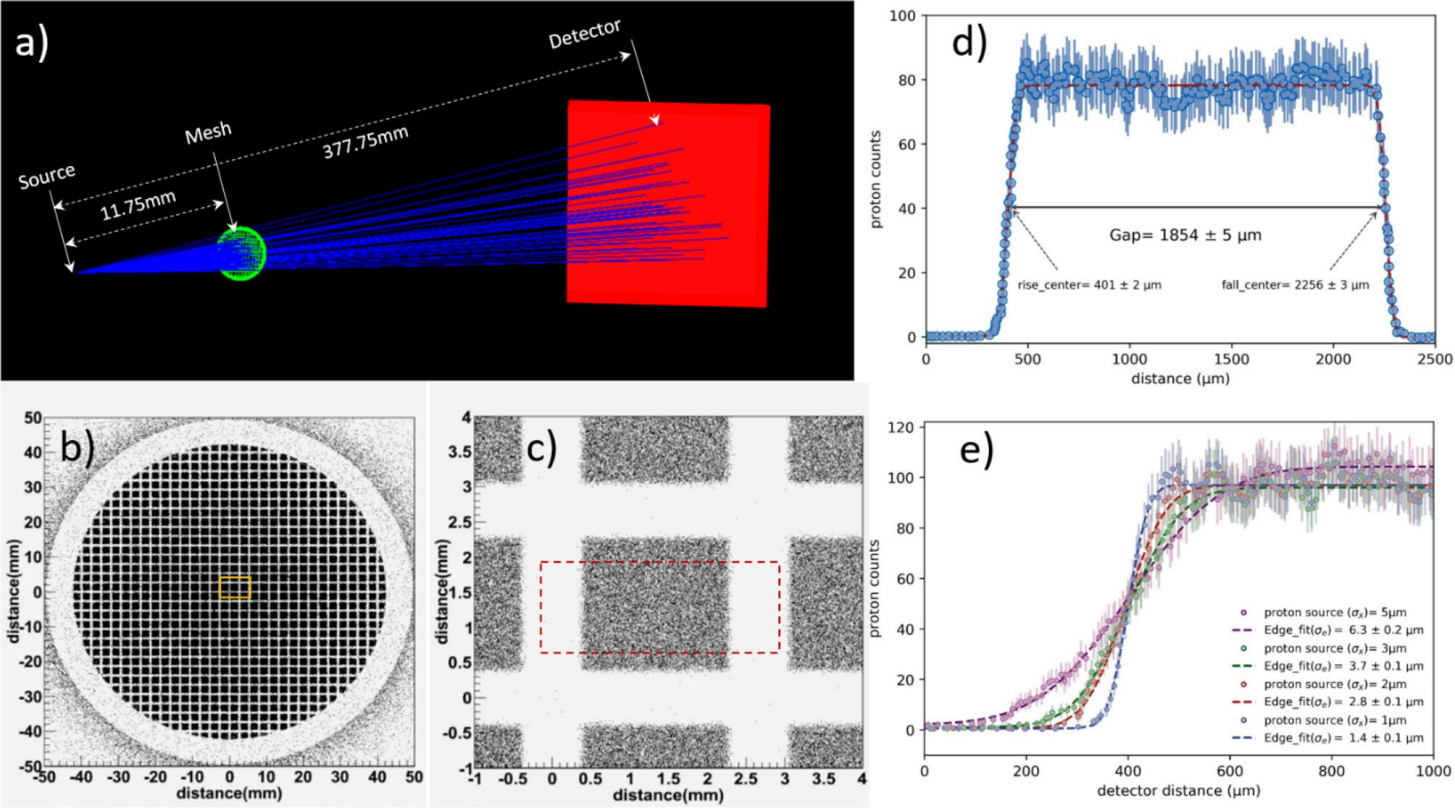
Monte-Carlo simulation for creating synthetic proton radiograph. (**a**) Geometry of the point-projection imaging simulation in Geant4. (**b**) Synthetic radiograph of 300LPI mesh grid generated by 0.5 MeV proton beam. (**c**) Enlarged image of the central mesh area, highlighted by Yellow Square in (**b**). (**d**) The spatial distribution of the proton count across the central mesh hole region, taken across the red square in (**c**). To determine the beginning and end location of the edged, the rising and falling slopes are fitted with the Gauss-error function. (**e**) The sharpness of the mesh edge is obtained by varying proton beam source sizes. To determine the edge sharpness, the proton pit distribution data are fitted with an error function.

The point-projection imaging of the mesh has been simulated by using the G4Beamline package, a Monte Carlo particle tracking tool, based on Geant4^52^. The synthetic radiograph of a 300 LPI Cu mesh, similar to use in the experiment (Fig. 1a), was generated by launching a 0.5 MeV proton beam, consisting of 10^7^ particles, with a divergence of 5-degree. The source size of the proton beam was considered as Gaussian distribution (${\sigma }_{x}= {\sigma }_{y})$ with different sizes varying from 1–10 µm. For the proton imaging, the geometry, size and location of the proton source, mesh (3.05 mm diameter, 300 LPI) and detector (100 mm × 100 mm) were kept the same as in the experiment, just to realize a similar magnification factor of 32.2 (Fig. 6a). The synthetic radiograph of the 300 LPI circular mesh, obtained by the 0.5 MeV proton beam is shown in Fig. 6b), which qualitatively reproduces the experimental mesh imaging picture (Fig. 2a). For detailed quantitative analysis, an enlarged region of the central mesh area is selected (Fig. 6c) and the corresponding proton count distribution across the central hole (encircled with red dotted square in Fig. 6c) is displayed in Fig. 6d. After fitting the rising and falling edges with the Gauss-error function, the 58 µm wide gap of the 300 LPI mesh turns out to be $1854 \pm 5$ µm (Fig. 6d) in the detector plane. The magnification factor of this point-projection imaging comes out to be $32.0 \pm 0.1,$ which is in good agreement with the experimentally measured average magnification factor of $\langle{M}_{exp}\rangle =31.7 \pm 0.5$. This indicates that our experimental conditions are close to the geometrical point-projection imaging. Furthermore, the effect of mesh edge sharpness is also shown (Fig. 6e), where the source size of the proton beam is varied. By comparing the experimental result ${\sigma }_{source}$ = 1.4 ± 0.1 µm (Fig. 3c), the best match can be found for proton beam of having source size of 1 µm for which of the edge ${\sigma }_{edge}=1.4 \,\upmu{\mathrm{m}}$ (Fig. 6e).

## Discussion

The few-cycle laser-foil interactions inherently facilitate the confining of plasma fields in both the space and time domain. In the spatial domain, these laser pulses under tight focusing conditions (F/2 OAP) with only a few 10’s of mJ laser energy do not support the presence of strong accelerating fields beyond a few micrometers. Similarly, in the time domain, the strong laser field, responsible for electron heating is present only for the duration of the laser pulse (12 fs), a time-frame too small for any significant plasma evolution involving ion motion. Furthermore, the use of high-temporal intensity contrast laser, where the target surface is not affected by the prepulse, can also suppress longitudinal and transverse pre-expansion of the electron cloud. In this study, the intensity in the pre-pulse region (up to 3.5 ps before the arrival of the main pulse) was kept below the level of 10^9^ W/cm^2^ (the laser intensity contrast of 10^–10^) and consequently ensured nearly pre-plasma free conditions for laser-foil interaction. Some previous experiments with simulations^25,26^ have indicated that high-contrast laser pulses tend to produce fast electrons with low angular divergence which in turn can help in confining the proton acceleration in a low angular cone. In future, it would be hence interesting to carry out a systematic study on the possibility of controlling the proton beam divergence and source size by changing laser intensity contrast and pulse duration.

## Conclusion

In summary, our experimental measurements have shown that the effective proton source size and the resultant spatial resolution is very similar to the laser focal spot size. Considering scalability to a kHz repetition rate, the demonstration of low-divergent proton source could be used for applications such as radiation therapy, warm dense matter and transmutation of nuclear waste, whereas the point like proton source feature can be applied for obtaining high-resolution static, dynamic radiographs, ion-beam lithography. Furthermore, these proton beams having low emittance can easily be transported and refocused over long distances with the help of proper beam-optics^21^ or can be post accelerated to higher energy by injecting in conventional accelerators^19,40^. In future, it would be interesting to see if the effective spatial resolution of the proton source can be further be reduced by using tighter laser focusing conditions (f/1 OAP).

## Methods

The experiment was performed with the SYLOS Experimental Alignment Laser (SEA Laser) at the ELI-ALPS facility in Szeged, Hungary^38^. A linearly polarized, NIR (840 nm), 12 fs, 35 mJ, laser pulse was focused with an f/2 off-axis parabolic (OAP) dielectric mirror on a 2 µm thick Al foil target at zero angle of incidence (Fig. 1a). The laser focal spot, measured with a microscopic objective (10$\times$, PAL-10-NIR, NA = 0.3) was found to be 2.9 µm $\times$ 3.5 µm (FWHM), containing 36% of total energy and therefore leading to the estimated peak intensity of 10^19^ W/cm^2^ (Fig. 1b). The temporal intensity contrast of the laser pulse, found to be 10^–10^ (3.5 ps before the arrival of the main pulse), limited the pre-pulse intensity to be below 10^9^ W/cm^2^ and consequently supported nearly pre-plasma free conditions for laser-foil interaction. To achieve the highest laser irradiance and shot-to-shot performance stability, each target were pre-positioned at the plane corresponding to the smallest laser focus with an accuracy of a few micrometers using the same microscopic objective back-illuminated with a white light source^53^. For kinetic energy spectrum of the forward accelerated ions, in the TPS, a small part (30 × 10^−9^ sr) of the total ion beam, sampled by a 200 µm diameter pinhole, were dispersed on a micro-channel-plate (MCP) detector based on their charge-to-mass ratio (q/m), by the parallel magnetic field (0.2 T) and electric field (3 kV/cm). The spatial characterization of the proton beam was carried out by projection imaging of a periodic mesh, 300 Lines-per-inch (LPI) onto a 1 mm thick CR-39 plate (10 cm $\times$ 10 cm) with a geometric magnification of 32.2. The distance between target and mesh was measured within uncertainty of ± 5 µm, by same microscope objective system, which was used for pre-positioning of the target surface. The distance between target and the CR-39 detector was measured with a metric ruler having precision of ± 0.5 mm. The CR-39 plate, after being exposed to the accelerated protons, was etched in a 6 N NaOH solution at a constant temperature of 70 °C for 60 min. After the etching process, the proton irradiated area has been recorded using an optical microscope (Zeiss Axio) with different magnifying objectives.

## Data Availability

All data generated or analysed during this study are included in this published article.
