# Author Correction: Low divergent MeV-class proton beam with micrometer source size driven by a few-cycle laser pulse

**DOI:** 10.1038/s41598-022-14559-2

**Published:** 2022-06-13

**Authors:** Prashant K. Singh, Parvin Varmazyar, Bence Nagy, Joon-Gon Son, Sargis Ter-Avetisyan, Karoly Osvay

**Affiliations:** 1 National Laser-Initiated Transmutation Laboratory, University of Szeged, 6720 Szeged, Hungary; 2 Department of Optics and Quantum Electronics, University of Szeged, 6720 Szeged, Hungary; 3 ELI-ALPS Research Institute, ELI-HU Non-Profit Ltd., Wolfgang Sandner utca 3, 6728 Szeged, Hungary

Correction to: *Scientific Reports* 10.1038/s41598-022-12240-2, published online 16 May 2022

The original version of this Article omitted an affiliation for Joon-Gon Son. The correct affiliations are listed below.

National Laser-Initiated Transmutation Laboratory, University of Szeged, 6720, Szeged, Hungary

ELI-ALPS Research Institute, ELI-HU Non-Profit Ltd., Wolfgang Sandner utca 3, 6728 Szeged, Hungary

In addition, the Acknowledgements section was incomplete.

“The project has been supported by the Ministry of Technology and Innovation (contract # IFHÁT/1039-4/2019-ITM_SZERZ) and by the National Research, Development, and Innovation Office through the National Laboratory program (contract # NKFIH-877-2/2020).”

now reads:

“The project has been supported by the Ministry of Technology and Innovation (contract # IFHÁT/1039-4/2019-ITM_SZERZ) and by the National Research, Development, and Innovation Office through the National Laboratory program (contract # NKFIH-877-2/2020). The authors are grateful for scientists and engineers of ELI-ALPS for helps in the experiment, as for Balazs Farkas for the loan of the vacuum chamber and various equipment, as well as for Adam Borzsonyi, Janos Csontos, Tamas Somoskoi, Laszlo Toth and Szabolcs Tóth for providing the laser beam for the experiment.”

Finally, the Funding section was incomplete.

“Open access funding provided by University of Szeged.”

now reads:

“Open access funding provided by University of Szeged. The ELI-ALPS project (GINOP-2.3.6-15-2015-00001) is supported by the European Union and co-financed by the European Regional Development Fund.”

The original Article has been corrected.

